# Explainability of a Deep Learning-Based Classification Model for Antineutrophil Cytoplasmic Autoantibody–Associated Glomerulonephritis

**DOI:** 10.1016/j.ekir.2024.11.005

**Published:** 2024-11-14

**Authors:** Maria A.C. Wester Trejo, Maryam Sadeghi, Shivam Singh, Naghmeh Mahmoodian, Samir Sharifli, Zdenka Hruskova, Vladimír Tesař, Xavier Puéchal, Jan Anthonie Bruijn, Georg Göbel, Ingeborg M. Bajema, Andreas Kronbichler

**Affiliations:** 1 Department of Pathology, Leiden University Medical Center, Leiden, The Netherlands; 2 Department of Medical Statistics, Informatics and Health Economics, Medical University of Innsbruck, Innsbruck, Austria; 3 Faculty of Computer Science, Otto von Guericke University, Magdeburg, Germany; 4 Department of Internal Medicine IV, Nephrology and Hypertension, Medical University of Innsbruck, Innsbruck, Austria; 5 Department of Nephrology, First Faculty of Medicine, Charles University and General University Hospital in Prague, Prague, Czechia; 6 Department of Internal Medicine, Hôpital Cochin, AP-HP, Paris, France; 7 Department of Pathology and Medical Biology, University Medical Center Groningen, Groningen, The Netherlands

**Keywords:** ANCA, artificial intelligence, histopathology, machine learning, vasculitis

## Abstract

**Introduction:**

The histopathological classification for antineutrophil cytoplasmic autoantibody (ANCA)–associated glomerulonephritis (ANCA-GN) is a well-established tool to reflect the variety of patterns and severity of lesions that can occur in kidney biopsies. It was demonstrated previously that deep learning (DL) approaches can aid in identifying histopathological classes of kidney diseases; for example, of diabetic kidney disease. These models can potentially be used as decision support tools for kidney pathologists. Although they reach high prediction accuracies, their “black box” structure makes them nontransparent. Explainable (X) artificial intelligence (AI) techniques can be used to make the AI model decisions accessible for human experts. We have developed a DL-based model, which detects and classifies the glomerular lesions according to the Berden classification.

**Methods:**

Kidney biopsy slides of 80 patients with ANCA-GN from 3 European centers, who underwent a diagnostic kidney biopsy between 1991 and 2011, were included. We also investigated the explainability of our model using Gradient-weighted Class Activation Mapping (Grad-CAM) heatmaps. These maps were analyzed by pathologists to compare the decision-making criteria of humans and the DL model and assess the impact of different training settings.

**Results:**

The DL model shows a prediction accuracy of 93% for classifying lesions. The heatmaps from our trained DL models showed that the most predictive areas in the image correlated well with the areas deemed to be important by the pathologist.

**Conclusion:**

We present the first DL-based computational pipeline for classifying ANCA-GN kidney biopsies as per the Berden classification. XAI techniques helped us to make the decision-making criteria of the DL accessible for renal pathologists, potentially improving clinical decision-making.

## Graphical abstract

**Figure:**
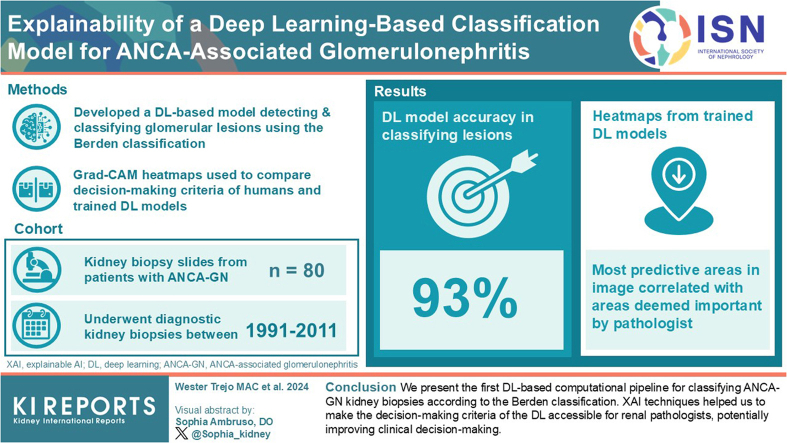

AI is moving into clinical pathology practice at an astounding speed. A central theme is how AI can be used as a decision support tool for pathologists, with the potential for gain in productivity and time saving. With its natural segmentation, kidney tissue forms an optimal subject for algorithms designed to recognize glomeruli, interstitial areas, and vessels.^1^^,^^2^

An ever-increasing number of studies demonstrate the effectiveness of DL models that can accurately perform the segmentation process of kidney tissue.^3^^,^^4^ Convolutional neural networks^5^ have been developed that can detect and classify renal structures and common lesions.^6^, ^7^, ^8^

Current AI applications in rare disease research are underrepresented so far.^1^ One such rare disease is ANCA-associated vasculitis, which is characterized by necrotizing small vessel vasculitis affecting multiple organ systems. Of all patients with systemic disease, 80% to 90% show kidney involvement in the form of ANCA-GN.^9^ Typically, by light microscopy, ANCA-GN is characterized by variable involvement of glomeruli, ranging from biopsies in which the majority of glomeruli are normal, to those in which most of the glomeruli are affected by crescents or chronic changes such as global sclerosis. In 2010, a histopathologic classification system for ANCA-GN was proposed, that was shown to reflect the variety and severity of glomerular lesions that can occur in ANCA-GN.^10^

Although algorithms can reach high prediction accuracies, their “black box” structure makes them nontransparent.^11^ A disadvantage is that the networks’ decisions are not easily interpretable by medical experts, and it is often unclear which information in the input data underlies the algorithms’ decisions. This necessitates the use of XAI, so that decisions made by AI models become accessible for verification by a human expert. One popular XAI method is Grad-CAM,^12^ which utilizes the gradients of the predicted class to generate a heatmap that indicates the most salient regions of an input image with regard to the model’s classification decision.

In this study, we focus on glomerular lesions of ANCA-GN and present the first DL-based computational pipeline for classifying kidney biopsies according to the Berden classification. In addition, we examined the explainability of our model using XAI techniques, to shed light on the “black box” of our trained DL classifier.

## Methods

### Image Data

Kidney biopsy slides of 80 patients with ANCA-GN, who underwent a diagnostic kidney biopsy between 1991 and 2011, were included. Sixty biopsies were used for the training of the models, and 20 different biopsies were used as a test set to validate our model. The biopsies were obtained from 3 European centers (Leiden University Medical Center, General University Hospital Prague, and Hôpital Cochin Paris) and were collected as part of a larger international cohort, used for an international validation study of the Berden classification.^13^ The biopsies were processed in a comparable way in these centers regarding embedding, cutting, and staining; and all had the same, excellent quality. For practical reasons, we used silver staining to train and test our model, because it was available for all biopsies. The glass slides were scanned using a whole-slide scanner (Phillips) at 40× magnification. In the whole-slide images (WSIs), the glomeruli were annotated and labelled jointly by IMB and MACWT as either “normal,” “sclerotic,” “crescentic,” or “abnormal-other,” thereby reaching an interobserver agreement of 100%. The publicly available, automated slide analysis platform annotation tool was used.^14^

### DL Models

**Figure 1:**
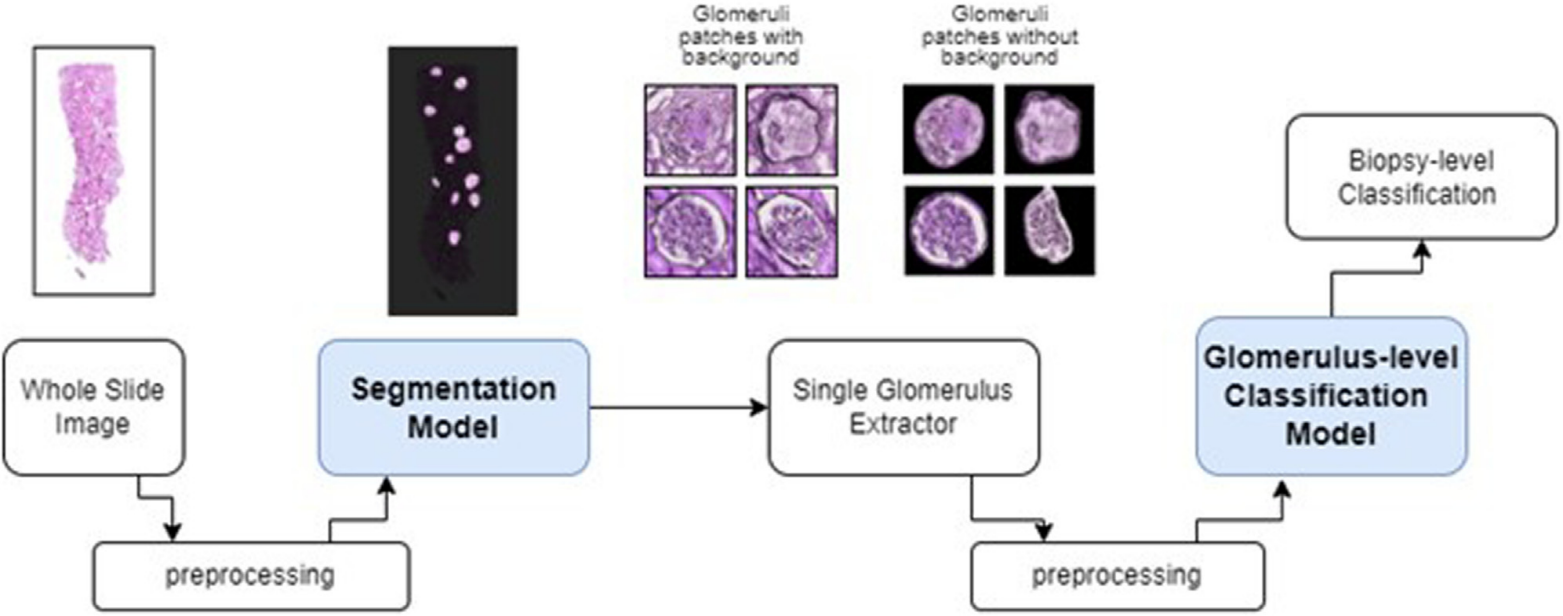
Schematic representation of the computational pipeline used for the analysis of whole-slide images (WSI). WSI from 60 biopsies were used for the training and validation of the model, after which the model was tested on a test set of 20 separate biopsies. The pipeline consists of 3 main stages, namely detection of all the glomeruli in the WSI, patch extraction containing these glomeruli, and glomerulus-level classification, which ultimately leads to biopsy-level classification. The glomeruli were classified into either “normal,” “sclerotic,” or “crescent.” If the model could not assign one of these categories to the glomerulus image with a confidence score of 0.8 or higher, the image fell into the category “abnormal–other.”

To analyze the WSIs, a computational pipeline was developed, as illustrated (Figure 1). The initial stage of the pipeline involved utilizing a segmentation model to detect and segment the glomeruli (glomeruli detection module). Subsequently, in the second step, patches containing glomeruli were extracted. Finally, in the last step, a glomeruli-level classification model was applied to classify the glomeruli into one of our predefined categories (glomeruli classification module).

### Downsampling

To detect glomeruli in a WSI, the image was downsampled by a factor of 4. The WSIs in our dataset had an average size of 12,000 × 16,000 pixels, which were downsampled to a size of 3000 × 4000 pixels to expedite computations in this stage. A sliding window approach was then employed to segment the entire WSI for glomerular structures, producing a slide-level segmentation of the WSI, which enabled detection of glomeruli. Based on the detected coordinates of each glomerulus in the WSI, a higher resolution image of the glomerular structure was cropped from a WSI that was downsampled only by a factor of 2. A glomerulus-level segmentation mask was generated using the same segmentation model. The glomeruli patches were then forwarded to the classification stage, along with their corresponding segmentation masks.

During the glomeruli detection step, WSIs were downsampled by a factor of 4, whereas a factor of 2 was used for the classification model. This difference in downsampling was intentional, because less detail is needed for glomeruli detection than for their classification. Downsampling is a common practice to reduce computational load; however, it was crucial to ensure that the resolution remained sufficient for accurate classification of glomerular structures.

The images used in the classification stage were resized to 224 × 224 pixels, retaining enough detail to preserve essential pathological features, thereby mitigating any potential negative impact on model performance. We also tested the classifier model on higher-resolution patches (456 × 456 pixels) during the testing phase. Because there was no significant difference in classification performance between the 2 resolutions, we opted for the smaller size to reduce computational complexity and focus on comparing other factors.

### Glomeruli Detection Module

The segmentation model used for detecting the glomeruli in the WSI images was based on a U-Net architecture^15^ that was pretrained on the ImageNet dataset.^16^ ImageNet is a large-scale dataset, primarily composed of natural images across various categories, not specific to medical or histological images. Pretraining on ImageNet enables the model to learn fundamental features such as edges and textures, which are transferable to medical images. The model was then trained using a supervised learning approach on a training set, which was generated based on the annotations provided by 2 experts. The resultant dataset contained 750 glomerular images and 750 images of nonglomerular regions, each with a size of 256 × 256 pixels. Data augmentation techniques were applied to the training set to increase the number of glomerular images. This is a common procedure to artificially increase the training set, making use of the existing data. It encompasses, for example, flipping, resizing, and adjusting the contrast of the images to increase the size and diversity of the training set.

### Glomeruli Classification Module

In the next stage, the glomeruli patches extracted by the glomeruli detection module were classified using the classification model. An EfficientNetB1 architecture^17^ was employed to classify the glomeruli into 1 of 3 categories: “normal,” “sclerotic,” and “crescent.” In selecting the architecture for our classification model, we opted for EfficientNet because of its advanced approach to scaling model dimensions—depth (d), width (w), and resolution (r)—using a compound coefficient (ϕ). Unlike traditional convolutional neural network architectures that require manual tuning of these parameters, EfficientNet's systematic scaling optimizes the allocation of computational resources, resulting in superior accuracy and efficiency. In pathological image analysis, multiple studies have utilized the EfficientNet model and achieved superior accuracy,^18^, ^19^, ^20^, ^21^, ^22^ making it particularly suitable for the complexity of glomerular lesion classification in ANCA-GN.

Out of the total of 80 biopsies, 60 biopsies containing 1127 images of glomeruli were used for the training and validation set. Twenty different biopsies with a total number of 388 glomeruli were used for the test set, thereby testing the generalizability. With regard to the different centers where the biopsies came from, these were randomly divided over the different data sets (data shuffling). We made sure that the distribution of the different classes was consistent in the training, validation, and test set. The images of the training dataset were categorized into crescentic (*n* = 533), sclerotic (*n* = 251), and normal (*n* = 343) classes. The “abnormal-other” class was not explicitly labeled in the training set but was generated during the model's inference stage. Glomeruli that did not achieve a confidence score of 0.8 or higher in being classified as sclerotic, normal, or crescentic were automatically assigned to the *“*abnormal-other” category. This approach allowed for the identification of ambiguous cases that do not clearly fall into the predefined categories, enhancing the model's robustness.

Because the primary focus of this study was to investigate the decision-making factors of our model, we tested 2 modifications that influence the performance of DL models, namely pretrained weights and the training data. Pretrained weights refer to the knowledge that the model has captured in its weights and biases through prior training on larger common datasets such as ImageNet. To examine the influence of pretrained weights, 2 models were trained. Model 1 (X1) was pretrained on the ImageNet dataset before being trained on our dataset, whereas Model 2 (X2) was pretrained on the ImageNet dataset and subsequently fine-tuned on a publicly available digital pathology dataset^23^ to enhance its knowledge of histopathological images before being trained on our own dataset.

To assess the influence of the training data, 2 identical training sets were created, with the only variation being that one dataset excluded the parts of the image outside the glomerular structure, utilizing the segmentation mask from the previous step (NoBG), whereas the other retained these parts (BG). Combinations of these modifications were tested to evaluate their effect on the model's performance and decision-making factors, resulting in 4 different models: X1_BG, X2_BG, X1_NoBG and X2_NoBG (Table 1).

**Table 1:** Average class accuracy for the different models

Pretrained dataset	Model architecture	Resolution	Dataset with background	Dataset without background
ImageNet	EfficientNetB1	224 × 224	Name: X1_BG ACA: 86%	Name: X1_NoBG ACA: 89%
ImageNet + DPD	EfficientNetB1	224 × 224	Name: X2_BG ACA: 91%	Name: X2_NoBG ACA: 93%

ACA, average class accuracy; BG, trained on glomerulus images with background; DPD, digital pathology dataset; NoBG, trained on glomerulus images without background; X1, pretrained on ImageNet only; X2, pretrained on ImageNet and DPD.

Summary of the effect of the different modifications on the performance and decision-making factors of the classification model. The table displays the average class accuracy as the performance metrics. The modifications tested include variations in the use of pretrained weights and the training data, as well as their combinations.

### XAI

Grad-CAM was employed for each of the trained networks in our study to explore the differences in decision-making factors resulting from the different training settings discussed in the previous section. This XAI technique utilizes the computed gradients of the model’s output (predicted class) to generate a heat-map, indicating the regions of importance in the input image.^12^ In our implementation of Grad-CAM, the heatmap generated by the technique was overlaid on the original input image to visually highlight the regions that were most relevant to the classification decision. By examining these heat-maps, we were able to gain insights into the decision-making process of our models and identify the regions that had the greatest influence on their predictions. Although Grad-CAM does not provide a complete explanation of the features that convolutional neural networks models use for classification, it can be a useful first step towards our understanding of how these models are able to classify glomerular lesions.

## Results

### DL Model

**Figure 2:**
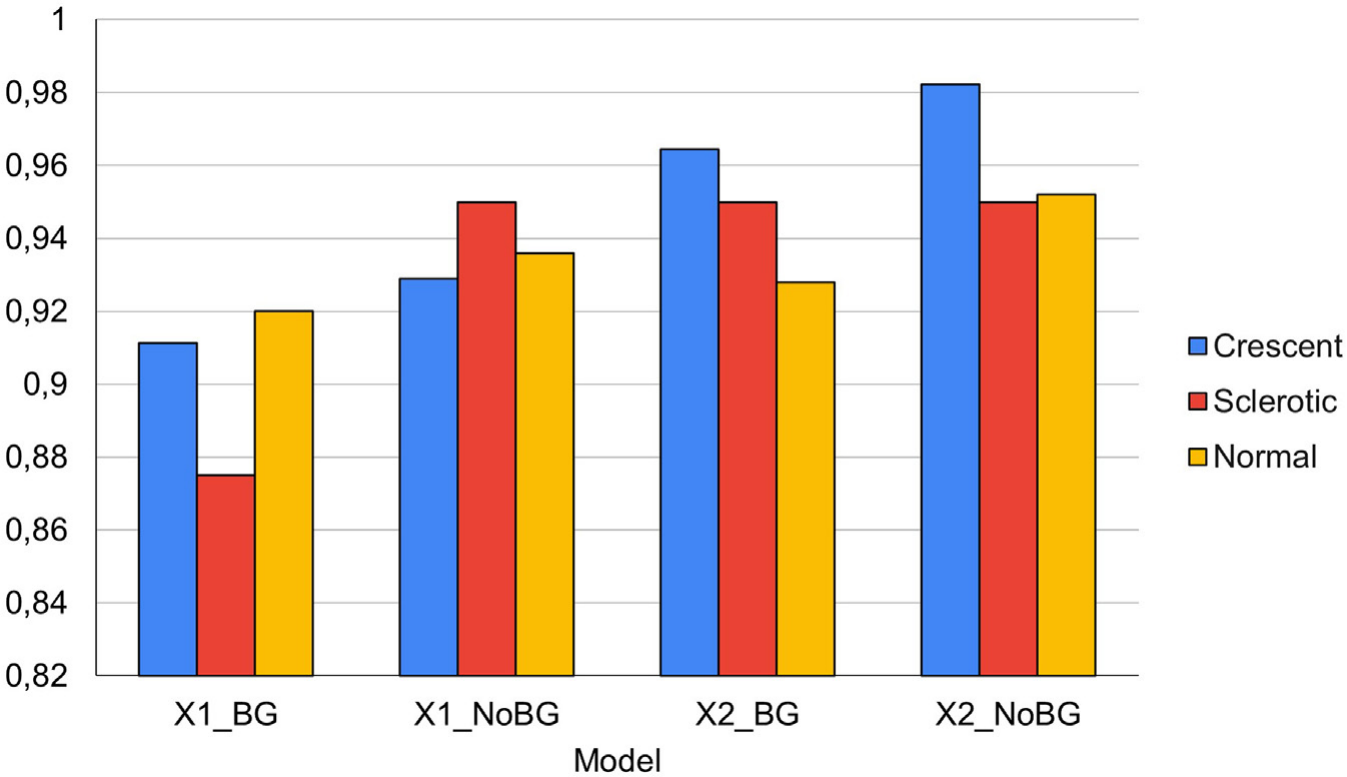
Performance of different glomerulus-level classification models on the test set of 20 whole-slide images with a total of 388 glomeruli. Accuracy is presented separately for each of the 3 categories. X2_NoBG achieved the highest accuracy of 93%. BG, trained on glomerulus images with background; DPD, digital pathology dataset; NoBG, trained on glomerulus images without background; X1, pretrained on ImageNet only; X2, pretrained on ImageNet and DPD.

The performance of the glomeruli segmentation model was assessed by testing it on 40 glomeruli, sampled from 10 different WSI that were not part of the training set. The model exhibited 100% accuracy in detecting the glomeruli. For pixel-wise segmentation, the model achieved an intersection over union score of 0.95. The performance of the glomerulus-level classification models was assessed by testing them on a separate test set containing 388 glomeruli sampled from 20 different WSI. In Figure 2, we illustrate the accuracy achieved by each model for the different categories. The numbers of glomeruli that were labelled differently by the model to the ground truth (false negatives) are shown in Table 2 for each category. The best performing model was X2_NoBG, pretrained on both the ImageNet and digital pathology dataset, and fine-tuned on our training set of glomeruli without background, achieving an accuracy of 93% (Table 1). It was observed that misclassified labels had an average confidence score of 0.81, whereas the correctly classified labels had an average confidence score of 0.95. The better the model gets, the average confidence score of wrongly classified images decreases. In model X2_NoBG, there are less false negatives than in the other models, and those false negatives have a lower confidence score. For more comprehensive metrics for model X2_NoBG refer to Table 3.

**Table 2:** Incorrectly classified glomeruli per category for the different models

Model	Number	Crescent	Sclerotic	Normal
		169	40	125
X1_BG	FN	15	5	10
	ACS	0.88	0.92	0.74
X1_NoBG	FN	12	2	8
	ACS	0.78	0.84	0.74
X2_BG	FN	6	2	9
	ACS	0.75	0.87	0.78
X2_NoBG	FN	3	2	6
	ACS	0.77	0.60	0.64

ACS, average confidence score; BG, trained on glomerulus images with background; DPD, digital pathology dataset; FN, false negatives; NoBG, trained on glomerulus images without background; X1, pretrained on ImageNet only; X2, pretrained on ImageNet and DPD.

For each model, the number of glomeruli that were classified differently to the ground truth (false negatives) are shown per category, as well as the corresponding average confidence score for these misclassifications. The better the model gets, the lower the confidence score for misclassified glomeruli.

**Table 3:** Performance metrics per category, based on the Berden histopathological classification

Category	Precision	Recall	F1-score	Support
Normal	0.80	0.95	0.87	125
Crescent	0.82	0.98	0.89	169
Sclerotic	0.97	0.95	0.96	40
Abnormal - other	0.00	0.00	0.00	0.00

DPD, digital pathology dataset.

Precision, recall, and F1-score for each category of glomeruli obtained from the best performing classification model, X2_NoBG. The model was trained on glomeruli patches without background (NoBG) and pretrained on ImageNet and DPD datasets (X2).

### XAI

**Figure 3:**
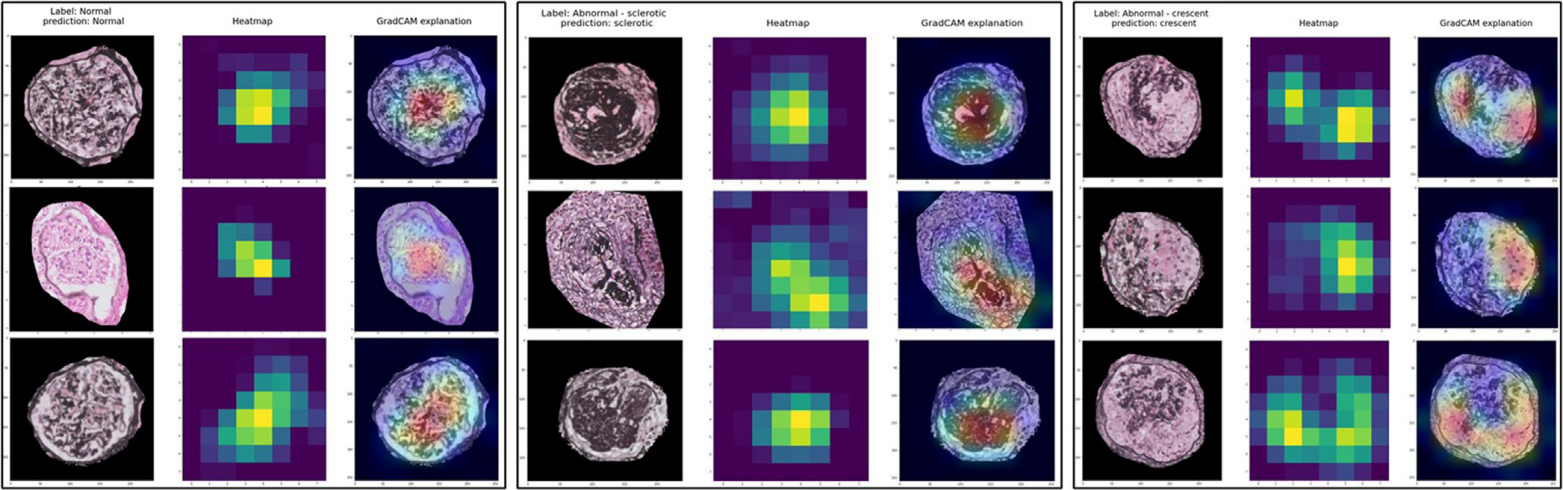
Examples of Grad-CAM heatmaps of glomeruli that were correctly classified by the model. The first panel shows 3 examples of normal glomeruli, the middle panel of globally sclerotic glomeruli, and the right panel of glomeruli with a crescent. Within each panel, the center column shows the heatmap, which is overlaid on the glomerulus image in the right column. The more yellow or red an area becomes, the more influential it was in the model’s prediction of the class.

To compare the decision-making criteria for classifying glomeruli of our DL models with those of a pathologist, heatmaps of all the individual glomeruli within the test set biopsies were analyzed. There were a total of 388 glomeruli in the test set, the heatmaps of which were analyzed using the 4 different models, leading to a total of 1552 heatmaps. Using Grad-CAM, we investigated whether the prediction maps highlighted regions that were diagnostically relevant. In glomeruli classified as normal, the open capillaries of the glomerular tuft were highlighted. In globally sclerosed glomeruli, the focus was on the sclerotic tuft. In the glomeruli containing a crescent, the focus was largely on the crescent itself, on areas with fibrinoid necrosis, and often on parts of the capillary tuft (Figure 3). These are the same features a pathologist focuses on during the diagnostic process.

### Misclassifications

In addition to investigating which areas of an image were the basis for classifying glomeruli, if they were correctly classified by the model, it is of interest to analyze the prediction maps of those glomeruli that were not classified in accordance with the ground truth. Those glomeruli that were labelled as normal by the pathologist and misclassified by the model, were all predicted to be crescentic by the model. The same is the case for sclerotic glomeruli. If misclassified, they were predicted to be crescentic. In line with this, of the total misclassified glomeruli that were originally labelled as crescentic, about half were predicted to be normal and the other half sclerotic. The model never predicted a normal glomerulus to be sclerotic, or vice versa, presumably because these are the most different morphologically.

As mentioned above, the model trained on images of glomeruli without background and the model pretrained on an extra dataset (“more experienced”) showed a higher prediction accuracy. We examined the heatmaps of the glomeruli that were misclassified by our 4 different models. When comparing the models trained on images of glomeruli with background versus without background, we found that the models mostly misclassified different glomeruli. The same was the case when comparing the models pretrained or not, on an extra dataset.

**Figure 4:**
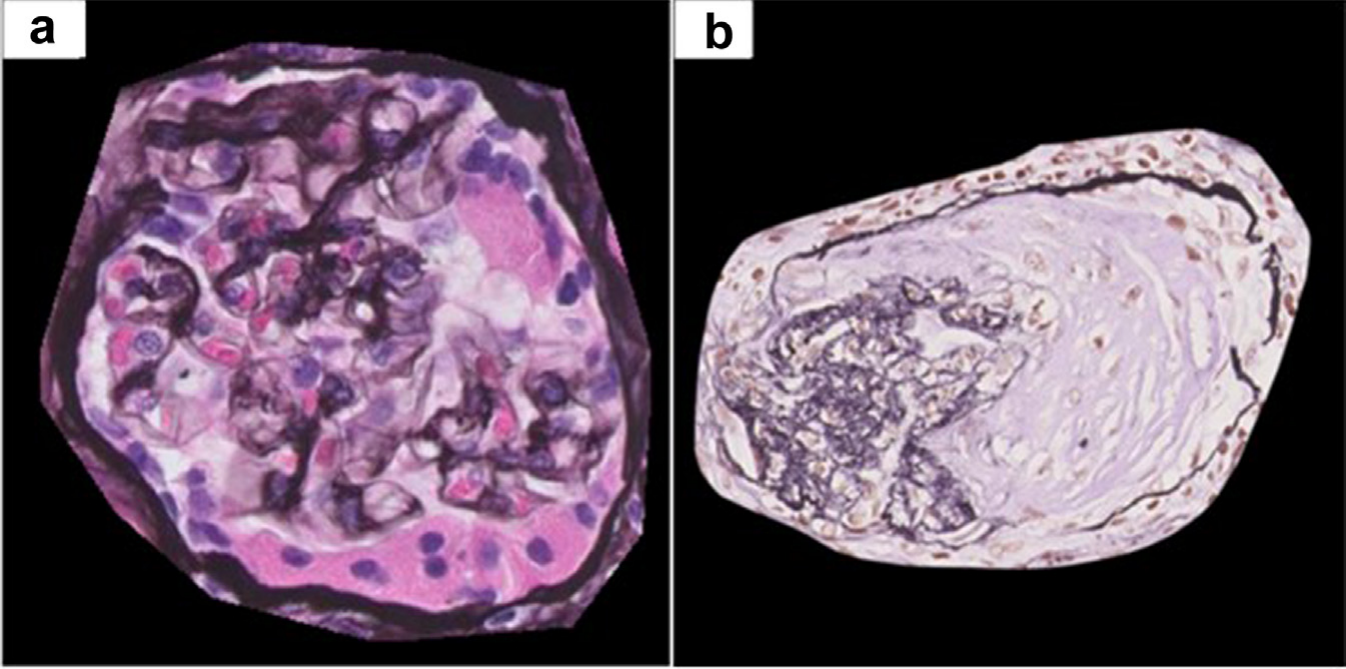
Examples of glomeruli incorrectly classified by the model, but understandably so. (a) Glomerulus with prominent parietal cells in Bowman’s space, erroneously classified as containing a crescent. (b) Globally sclerotic glomerulus erroneously classified as containing a crescent, which it may have contained at a previous disease stage.

Interestingly, in a proportion of the misclassified glomeruli, the prediction of the model can be understood when taking a look at the heatmaps. For example, Figure 4a shows a glomerulus with very prominent parietal cells lining Bowman’s capsule, based on which the model classified the glomerulus as crescentic. Regarding this as a crescent is a misinterpretation that is often seen in young trainees. Similarly, the sclerotic glomerulus in Figure 4b was predicted to be crescentic by the model, and it may have well contained a crescent before becoming sclerosed.

## Discussion

Kidney biopsies are the gold standard for predicting prognosis in ANCA-GN. Evaluating a kidney biopsy, which calls for assessing several modalities, is often a complex and time-consuming process characterized by significant interobserver variability. In ANCA-GN, interobserver variability was first described in 1996.^24^ More recently, we reported an interobserver variation for the Berden classification and found only moderate agreement (kappa score 0.56).^13^ AI models can help overcome this problem.

We present the first DL-based pipeline that can classify kidney biopsies of patients with ANCA-GN as per the Berden histopathological classification. The studies on DL models in nephropathology published so far, mainly concern segmentation and focus mostly on transplant biopsies. Ginley *et al.*^25^ were the first to publish a DL-model for a native kidney disease, namely diabetic kidney disease. The model classifies biopsies following the Tervaert pathologic classification of diabetic kidney disease.^26^ Recently, a model was published that can distinguish between minimal change disease, membranous nephropathy, and thin basement membrane nephropathy, although with relatively low accuracy.^27^ In addition, Kers *et al.*^28^ created a DL-based model that classifies kidney transplant biopsies into 1 of 3 classes: normal, rejection, or other diseases.

Our model classifies glomeruli as either normal, crescentic, globally sclerotic, or abnormal-other. This forms the basis for assigning 1 of the 4 Berden classes to the biopsy (focal, crescentic, sclerotic, or mixed), which in turn is associated with kidney outcome.^10^ Our model obtained a high accuracy of 93% for classification at the glomerular level, which is higher than the accuracies reported in the classification systems for diabetic kidney disease^25^ and transplant biopsies.^28^ We found that removing the background area surrounding glomeruli improved accuracy and further achieved a higher accuracy by training the model on a publicly available image dataset, before training it on our ANCA-GN cohort.

A study on segmentation of histologic structures showed the best concordance between the model segmentation and the pathologists’ annotations for Periodic Acid Schiff stains, whereas silver stains yielded the worst performance.^6^ Our model was trained on a single silver stain per patient, which did not cause a problem with regard to segmentation, because the model achieved 100% accuracy in recognizing glomeruli. With regard to classification, however, our model could potentially be improved by training on different stains in addition to silver, because different lesions may be highlighted in a stain-specific way. Interestingly, research is being done regarding ”virtual staining,” which generates various types of histological stains from images of unstained samples.^29^

DL-based classification models for pathology will soon be at a stage where they can be implemented in clinical practice. Possible limitations for implementation may be the transparency and interpretability of these models, referred to as the “black box” phenomenon.^11^ Here, we used XAI techniques to visualize the areas within the images, that were most predictive for the classification decision. We found that the model mostly focused on the same areas that a pathologist focuses on when evaluating a biopsy. In case of misclassification, it was comprehensible in a proportion of those glomeruli, because the most predictive areas showed changes that resembled the lesions the model predicted the image to have. In addition to making the diagnostic process of DL models more interpretable and increase trust, XAI techniques may be useful for improving a pathologist’s efficiency. It may be used to highlight the areas in a biopsy that are most relevant for reaching a diagnosis.

One of the limitations of our study is that we trained our model on images of glomeruli only, because these form the basis for the Berden classification. It is known, however, that tubulointerstitial parameters are of prognostic value, but show a considerable amount of interobserver variability.^10^^,^^13^ Incorporating these parameters in our model might improve its prognostic value. Another limitation is that the annotations were provided by only 2 pathologists. In addition, because ANCA-GN is a rare disease, we had to develop our model on a relatively low number of cases, which is another limitation of our study. To provide a more robust algorithm, the model would need to be trained on a larger dataset, where different types of scanners and more than 1 staining methods are used. If the evaluation of these biopsies were performed by a larger group of nephropathologists, the model would reflect the consensus of this group. Ultimately, a shift to DL models that incorporate not only histopathological information, but also clinical and genomic information would be desirable for optimal prognostication and individualized therapies.

In conclusion, we present the first DL-based histopathologic classification model for ANCA-GN. This model is an automated version of the Berden histopathological classification; however, going forward, incorporation of kidney outcome data in this model could refine and improve the prognostic value of the classification system. In addition, we used XAI techniques to illuminate the “black box” of the model, improving its interpretability. This can build trust and accelerate the implementation of AI models in clinical practice. It can also provide a possible support system in the evaluation of kidney biopsies.

## Disclosure

VT reports consulting fees from AstraZeneca, Boehringer Ingelheim, Calliditas Therapeutics, Novartis, Omeros, Otsuka, Travere Therapeutics, and Vera Therapeutics; payment or honoraria from Calliditas Therapeutics, GlaxoSmithKline, and Travere Therapeutics and participation on a data safety monitoring board or advisory board for Alexion, AstraZeneca, Bayer, Boehringer Ingelheim, Novartis, Omeros, Otsuka, Travere Therapeutics, and Vera Therapeutics. XP reports consulting fees from Argenx, GlaxoSmithKline, and Boehringer Ingelheim and travel support from AstraZeneca. IMB reports educational grants from CSL Vifor; consulting fees from Boehringer Ingelheim, GlaxoSmithKline, Novartis, Catalyst Biosciences, Toleranzia, Vera, and Hansa Biopharma and is the founder and owner of BiPath. AK reports unrestricted research grant support from CSL Vifor; consulting or speaking fees from AstraZeneca, Catalyst Biosciences, CSL Vifor, Delta4, GlaxoSmithKline, Miltenyi Biotech, Novartis, Roche, and Walden Biosciences, and received travel support from Otsuka and AstraZeneca. All the other authors declared no competing interests.

## Supplementary Material

Supplementary File (PDF) STROBE Checklist.
