# Supplementary material for: Explainability of a Deep Learning-Based Classification Model for Antineutrophil Cytoplasmic Autoantibody–Associated Glomerulonephritis
Source: Kidney Int Rep. 2024 Nov 14;10(2):457–65. doi: 10.1016/j.ekir.2024.11.005 (PMC11843110; doi:10.1016/j.ekir.2024.11.005)
Supplement: Supplementary File (PDF) — STROBE Checklist. [file mmc1.pdf]

STROBE Statement—checklist of items that should be included in reports of observational studies

	Item No.	Recommendation	Page No.	Relevant text from manuscript
**Title and abstract**	1	(*a*) Indicate the study’s design with a commonly used term in the title or the abstract	3	
		(*b*) Provide in the abstract an informative and balanced summary of what was done and what was found	3	
**Introduction**				
Background/rationale	2	Explain the scientific background and rationale for the investigation being reported	4, 5	
Objectives	3	State specific objectives, including any prespecified hypotheses	5	First DL-based Berden classification system, shed light on the ‘black box’
**Methods**				
Study design	4	Present key elements of study design early in the paper ‘	6	
Setting	5	Describe the setting, locations, and relevant dates, including periods of recruitment, exposure, follow-up, and data collection	6	‘Kidney biopsy slides of 80 patients with ANCA-GN, who underwent a diagnostic kidney biopsy between 1991 and 2011, were included. The biopsies were obtained from three European centers (Leiden University Medical Center, General University Hospital Prague and Hôpital Cochin Paris) and were collected as part of a larger international cohort, used for an international validation study of the Berden classification.’[13]
Participants	6	(*a*) *Cohort study*—Give the eligibility criteria, and the sources and methods of selection of participants. Describe methods of follow-up		

		Case-control study—Give the eligibility criteria, and the sources and methods of case ascertainment and control selection. Give the rationale for the choice of cases and controls<br>*Cross-sectional study*—Give the eligibility criteria, and the sources and methods of selection of participants	7	Please refer to reference 13
		(*b*) *Cohort study*—For matched studies, give matching criteria and number of exposed and unexposed<br>*Case-control study*—For matched studies, give matching criteria and the number of controls per case		
Variables	7	Clearly define all outcomes, exposures, predictors, potential confounders, and effect modifiers. Give diagnostic criteria, if applicable	6	In the whole-slide images (WSIs), the glomeruli were annotated and labelled jointly by IMB and MWT as either ‘normal’, ‘sclerotic’, ‘crescentic’ or ‘abnormal-other’
Data sources/ measurement	8*	For each variable of interest, give sources of data and details of methods of assessment (measurement). Describe comparability of assessment methods if there is more than one group	6	“
Bias	9	Describe any efforts to address potential sources of bias	9	‘pretrained weights’
Study size	10	Explain how the study size was arrived at	6	The biopsies were obtained from three European centers (Leiden University Medical Center, General University Hospital Prague and Hôpital Cochin Paris) and were collected as part of a larger international cohort, used for an international validation study of the Berden classification.[13]

Continued on next page

Quantitative variables	11	Explain how quantitative variables were handled in the analyses. If applicable, describe which groupings were chosen and why	8	'Out of the total of 80 biopsies, 60 biopsies containing 1127 images of glomeruli were used for the training and validation set; 20% of this data was used for the validation set during training. 20 different biopsies with a total number of 388 glomeruli were used for the test set.'
Statistical methods	12	(*a*) Describe all statistical methods, including those used to control for confounding		
		(*b*) Describe any methods used to examine subgroups and interactions	N/A	
		(*c*) Explain how missing data were addressed	N/A	
		(*d*) *Cohort study*—If applicable, explain how loss to follow-up was addressed<br>*Case-control study*—If applicable, explain how matching of cases and controls was addressed<br>*Cross-sectional study*—If applicable, describe analytical methods taking account of sampling strategy		
		(*e*) Describe any sensitivity analyses	N/A	
**Results**				
Participants	13*	(a) Report numbers of individuals at each stage of study—eg numbers potentially eligible, examined for eligibility, confirmed eligible, included in the study, completing follow-up, and analysed	8	
		(b) Give reasons for non-participation at each stage	N/A	
		(c) Consider use of a flow diagram		
Descriptive data	14*	(a) Give characteristics of study participants (eg demographic, clinical, social) and information on exposures and potential confounders	6	Please refer to reference 13
		(b) Indicate number of participants with missing data for each variable of interest	N/A	
		(c) *Cohort study*—Summarise follow-up time (eg, average and total amount)		
Outcome data	15*	*Cohort study*—Report numbers of outcome events or summary measures over time		
		Case-control study—Report numbers in each exposure category, or summary measures of exposure		
		Cross-sectional study—Report numbers of outcome events or summary measures	24-26	Table 1, 2 and 3

Main results	16	(*a*) Give unadjusted estimates and, if applicable, confounder-adjusted estimates and their precision (eg, 95% confidence interval). Make clear which confounders were adjusted for and why they were included	24-25	Table 1 and 2
		(*b*) Report category boundaries when continuous variables were categorized	N/A	
		(*c*) If relevant, consider translating estimates of relative risk into absolute risk for a meaningful time period	N/A	
Other analyses	17	Report other analyses done—eg analyses of subgroups and interactions, and sensitivity analyses	N/A	
**Discussion**				
Key results	18	Summarise key results with reference to study objectives	14,15	'Our model obtained a high accuracy of 93% for classification at the glomerular level, which is higher than the accuracies reported in the classification systems for DKD[25] and transplant biopsies.[28] We found that removing the background area surrounding glomeruli improved accuracy and achieved a higher accuracy by training the model on a publicly available image dataset, before training it on our ANCA-GN cohort.' 'Here, we used XAI techniques to visualize the areas within the images that were most predictive for the classification decision. We found that the model mostly focused on the same areas that a pathologist focuses on when evaluating a biopsy. In case of

				misclassification, it was comprehensible in a proportion of those glomeruli, as the most predictive areas showed changes that resembled the lesions the model predicted the image to have.’
Limitations	19	Discuss limitations of the study, taking into account sources of potential bias or imprecision. Discuss both direction and magnitude of any potential bias	15,16	‘One of the limitations of our study is that we trained our model on images of glomeruli only, as these form the basis for the Berden classification. It is known, however, that tubulointerstitial parameters are of prognostic value, but show a considerable amount of interobserver variability.10,13 Incorporating these parameters in our model might improve its prognostic value. Another limitation is that the annotations were provided by only two pathologists. Additionally, because ANCA-GN is a rare disease, we had to develop our model on a relatively low number of cases, which is another limitation of our study.’
Interpretation	20	Give a cautious overall interpretation of results considering objectives, limitations, multiplicity of analyses, results from similar studies, and other relevant evidence	14-16	
Generalisability	21	Discuss the generalisability (external validity) of the study results	16	‘To provide a more robust algorithm, the model would need to

				be trained on a larger dataset, where different types of scanners and more than one staining are used. If the evaluation of these biopsies would be performed by a larger group of nephropathologists, the model would reflect the consensus of this group. Ultimately, a shift to DL models that incorporate not only histopathological information, but also clinical and genomic information, would be desirable for optimal prognostication and individualized therapies.’
**Other information**				
Funding	22	Give the source of funding and the role of the funders for the present study and, if applicable, for the original study on which the present article is based	17	

*Give information separately for cases and controls in case-control studies and, if applicable, for exposed and unexposed groups in cohort and cross-sectional studies.

Note: An Explanation and Elaboration article discusses each checklist item and gives methodological background and published examples of transparent reporting. The STROBE checklist is best used in conjunction with this article (freely available on the Web sites of PLoS Medicine at http://www.plosmedicine.org/, Annals of Internal Medicine at http://www.annals.org/, and Epidemiology at http://www.epidem.com/). Information on the STROBE Initiative is available at www.strobe-statement.org.
